# Resectable left lower lobe non–small cell lung cancer with lymph node metastasis is related to unfavorable outcomes

**DOI:** 10.1186/s40880-015-0069-8

**Published:** 2016-01-06

**Authors:** Wen-Feng Ye, Xuan Xie, Hong Yang, Kong-Jia Luo, Qian-Wen Liu, Yu-Zhen Zheng, Jun-Ye Wang

**Affiliations:** State Key Laboratory of Oncology in South China, Collaborative Innovation Center for Cancer Medicine, Sun Yat-sen University Cancer Center, Guangzhou, 510060 Guangdong P.R. China; Department of Clinical Nutrition, Sun Yat-sen University Cancer Center, Guangzhou, 510060 Guangdong P.R. China; Department of Thoracic Surgery, Sun Yat-sen Memorial Hospital, Sun Yat-sen University, Guangzhou, Guangdong 510120 P.R. China; Department of Thoracic Oncology, Sun Yat-sen University Cancer Center, Guangzhou, Guangdong 510060 P.R. China

**Keywords:** Lung cancer surgery, Lobectomy, Tumor, Outcomes, Lymph node

## Abstract

**Background:**

Despite numerous previous studies, the consideration of tumor location as a prognostic factor in resectable non–small cell lung cancer (NSCLC) remains controversial. The present study analyzed the association between tumor location and clinical outcome in patients with resectable NSCLC who had undergone lobectomy with systematic lymphadenectomy and who had presented with varying nodal statuses.

**Methods:**

The data from a cohort of 627 eligible patients treated in Sun Yat-sen University Cancer Center between January 2000 and December 2008 were retrospectively collected, and the nodal statuses of patients with different tumor locations were compared. Cox proportional hazards regression model was used to determine the independent factors related to cancer-specific survival (CSS).

**Results:**

Multivariate analysis demonstrated that left lower lobe (LLL) tumors [hazard ratio (HR): 1.465, 95% confidence interval (CI) 1.090–1.969, *P* = 0.011], lymph node metastasis (HR: 2.742, 95% CI 2.145–3.507, *P* < 0.001), and a tumor size of >4 cm (HR: 1.474, 95% CI 1.151–1.888, *P* = 0.002) were three independent prognosticators in patients with resectable NSCLC. However, LLL tumors were associated only with CSS in node-positive patients (HR: 1.528, 95% CI 1.015–2.301, *P* = 0.042), and a tumor size of >4 cm was the only independent risk predictor in the node-negative subgroup (HR: 1.889, 95% CI 1.324–2.696, *P* < 0.001).

**Conclusions:**

Tumor location is related to the long-term CSS of NSCLC patients with lymph node metastasis. LLL tumors may be upstaged in node-positive patients to facilitate an optimal treatment strategy.

## Background

Lung cancer is the first leading cause of cancer death in China [1] and around the world [2]. Despite the incorporation of standard multimodality therapies tailored by the staging system, non–small cell lung cancer (NSCLC) remains an aggressive disease with a dismal prognosis [3–5], highlighting the importance of seeking other prognostic factors and applying adjuvant or biological therapy for patients with poor prognosis.

Although tumor size and invasiveness have been used for many years in determining the T category of various cancers, tumor location is undervalued. Although this factor is not incorporated in the seventh edition of the International Association for the Study of Lung Cancer tumor-node-metastasis (TNM) staging system [6], tumor location has long been suggested as a potential, albeit controversial, prognostic factor [7–11]. Previous investigators have found that non-upper lobe tumor location was an adverse prognostic factor for patients with stage I or III NSCLC [8–11]. However, a recent study by Puri et al. [12] indicated that tumor location does not predict survival of patients with pathologic stages I and II NSCLC. Kudo et al. [13] also reported that tumor location influences only the long-term survival of NSCLC patients with lymph node metastasis. In addition, Whitson et al. [14] found that, although tumor location is associated with lymph node yield, it is not an independent predictor of survival.

The conflicting results from previous studies may be partially due to the variation in the method by which the tumor locations, such as non-upper and upper, right- and left-sided, and left lower lobe (LLL) and non-LLL tumor, were compared. Moreover, various surgical approaches, including pneumonectomy, wedge resection, and segmental resection, were used in their studies, possibly influencing the survival of patients with resectable NSCLC [15, 16]. In the present study, we therefore selected NSCLC patients who had undergone lobectomy followed by systematic lymph node dissection and explored whether tumor lobe location was an independent prognostic factor for cancer-specific survival (CSS) based on nodal status.

## Patients and methods

### Patient selection

This study was approved by the Ethics Committee of the Sun Yat-sen University Cancer Center and was conducted using a database of 1578 consecutive patients who had undergone surgery for NSCLC at the Department of Thoracic Oncology between January 2000 and December 2008. Eligible patients included those with histologically proven NSCLC and had undergone complete resection, such as lobectomy (including bilobectomy) and systematic lymph node dissection (R0 resection). Patients who had undergone wedge resection, sleeve resection, segmentectomy, and pneumectomy and those with mediastinal lymph nodes (MLNs) being dissected at less than three stations were not included in the study. Patients who met any of the following criteria were also excluded: tumor located at a fissure and incorporating more than one lobe, the presence of distant metastasis before operation, neoadjuvant therapy before operation, previous or concurrent malignancy, or postoperative death within 1 month. All patients provided written informed consent before surgery.

### Therapeutic process

Thoracotomy or video-assisted thoracoscopy were performed. The tumor location was marked during surgery as the right upper lobe (RUL), right non-upper lobe (RMLL), left upper lobe (LUL), or LLL. Following fixation in 10% neutral buffered formalin for approximately 24 h at room temperature, the tumor size was measured and therefore the maximum diameter of the tumor was recorded. At least 6 stations of lymph nodes and no less than three stations of MLNs were dissected with the guidance of the Mountain/Dresler lymph node map [17]. The mediastinal lymphadenectomy procedure was performed in accordance with the approach described in Allen’s study [18]. Each patient was staged according to the seventh edition of TNM classification [6].

Four to six cycles of cisplatin-based adjuvant chemotherapy were recommended to high-risk N0 and node-positive patients undergoing surgery after 2004.

### Follow-up

Follow-up was generally carried out 1 month after surgery, every 6 months for the first 3 years, and annually thereafter. The routine examinations included a physical examination, chest radiography, and ultrasound of the abdomen (including the liver, pancreas, spleen, kidney, and adrenal glands). Chest and upper abdominal computed tomography and brain magnetic resonance imaging (MRI) were conducted every year. Bone scan, bronchoscopy, and vertebral MRI were performed only in symptomatic patients. Patients who were lost to follow-up were censored at the last time of contact. The median length of follow-up for surviving patients was 84 months (range 1–163 months).

### Statistical analysis

All statistical analyses were performed with SPSS version 16.0 (SPSS, Chicago, IL, USA). Categorical variables were compared by using χ^2^ test or Fisher’s exact test, when applicable. The differences in the numerical variables between the two groups were analyzed using the Mann-Whitney *U* test. Univariate and multivariate survival analyses with calculation of the hazard ratio (HR) were performed by using Cox proportional hazards regression model. Potential prognostic factors with a *P* value of less than 0.10 were entered into multivariate analyses. A stepwise forward procedure was used to derive a final model of the variables. The CSS was defined as the duration from the date of the operation to either the date of death from NSCLC or the last follow-up (August 2, 2013). A significant difference was declared if the *P* value from a two-tailed test was less than 0.05.

## Results

### Demographic characteristics

A total of 627 patients were included in this study. Their clinicopathologic characteristics are summarized in Table 1. Histologically, 363 tumors were classified as adenocarcinoma, and 191 tumors were classified as squamous cell carcinoma; the remaining 73 tumors were miscellaneous types, including adenosquamous carcinoma, large cell carcinoma, and carcinoid carcinoma. A total of 587 patients underwent a single lobectomy; the remaining 40 patients underwent bilobectomy, including 8 lesions in the RUL, 6 lesions in the right median lobe, and 26 lesions in the right lower lobe.

**Table 1:** Clinicopathologic characteristics of the entire non–small cell lung cancer (NSCLC) patient cohort (*n* = 627)

Characteristic	No. of patients
Sex	
Men	432
Women	195
Age (years)^a^	59 (23, 79)
Tumor location	
LUL	123
LLL	107
RUL	208
RMLL	189
T category	
TI	90
T2	462
T3	75
N category	
N0	390
N1	66
N2	171
Pathology	
AC	363
SCC	191
Others	73
Histology grade	
G1	103
G2	206
G3	318
Tumor size (cm)^a^	3.5 (1, 13)
Pleural invasion	
No	91
Yes	536
Resection	
Lobectomy	587
Bilobectomy	40
Adjuvant chemotherapy	
Yes	228
No	399
No. of dissected MLNs^a^	7 (3, 44)

*LUL* left upper lobe, *LLL* left lower lobe, *RUL* right upper lobe, *RMLL* right non-upper lobe, *AC* adenocarcinoma, *SCC* squamous cell carcinoma, *MLNs* mediastinal lymph nodes

^a^The values of these items are presented as median followed by the range in the parentheses

### Tumor location and CSS in the entire cohort

During the follow-up period, 275 patients died, including 12 from non-cancer-related causes, such as myocardial infarction, cerebral infarction, pneumonia, and vehicular accidents. The 5-year CSS rate of the entire cohort was 61.6%. The relationship between clinicopathologic characteristics and CSS is shown in Table 2. Univariate survival analysis indicated that tumors located in the non-upper lobe (*P* = 0.013), LLL tumors (*P* = 0.007), tumors with advanced T category (*P* = 0.001), lymph node metastasis (*P* < 0.001), a tumor size of >4 cm (*P* < 0.001), the presence of pleural invasion (*P* = 0.015), a high histology grade (*P* = 0.006), and bilobectomy (*P* = 0.044) were risk factors significantly associated with CSS. Multivariate analysis demonstrated that LLL (HR: 1.465, 95% confidence interval CI 1.090–1.969, *P* = 0.011), lymph node metastasis (HR: 2.742, 95% CI 2.145–3.507, *P* < 0.001), and a tumor size of >4 cm (HR: 1.474, 95% CI 1.151–1.888, *P* = 0.002) were independently associated with dismal CSS in patients with resected NSCLC.

**Table 2:** Univariate and multivariate analysis of prognostic variables for patients with resected NSCLC (*n* = 627)

Characteristic	Univariate analysis		Multivariate analysis	
	HR (95% CI)	*P* ^a^	HR (95% CI)	*P* ^a^
Sex (men vs. women)	0.827 (0.633–1.080)	0.162	–	–
Age (≤60 years vs. >60 years)	1.076 (0.844–1.371)	0.556	–	–
Tumor location				
(Left vs. right lobe)	0.904 (0.705–1.160)	0.428	–	–
(Upper vs. lower lobe)	1.362 (1.068–1.735)	0.013	–	–
(Non-LLL vs. LLL)	1.493 (1.115–1.998)	0.007	1.465 (1.090–1.969)	0.011
T category (T1 vs. T2 vs. T3)	1.492 (1.173–1.898)	0.001	–	–
N category (N0 vs. N1–2)	2.749 (2.152–3.512)	0.000	2.742 (2.145–3.507)	<0.001
Pathology (AC vs. SCC vs. others)	1.103 (0.927–1.312)	0.269	–	–
Histology grade (G1 vs. G2 vs. G3)	1.267 (1.071–1.499)	0.006	–	–
Tumor size (≤4 vs. >4 cm)	1.617 (1.266–2.065)	0.000	1.474 (1.151–1.888)	0.002
Pleural invasion (no vs. yes)	1.627 (1.099–2.408)	0.015	–	–
Resection (lobectomy vs. bilobectomy)	1.555 (1.013–2.386)	0.044	–	–
Adjuvant chemotherapy (yes vs. no)	0.828 (0.644–1.065)	0.141	–	–

*LLL* left lower lobe, *non-LLL* other lobes, *AC* adenocarcinoma, *SCC* squamous cell carcinoma, *HR* hazard ratio, *CI* confidence interval

^a^Cox proportional hazards regression analysis (forward stepwise)

### Tumor location and CSS based on nodal status

**Fig. 1:**
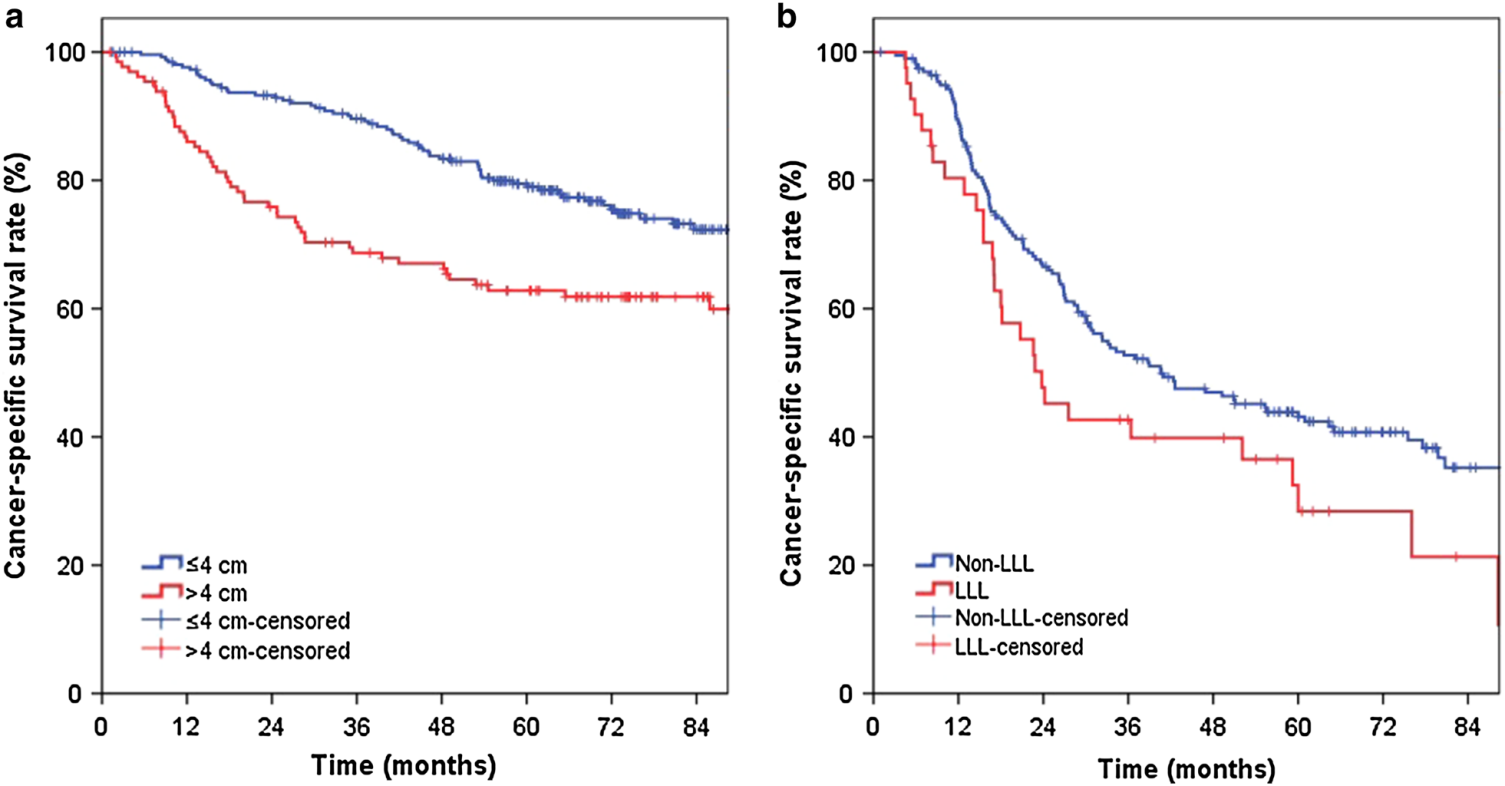
Kaplan–Meier survival analysis in patients with resectable non–small cell lung cancer (NSCLC). **a** The relationship between tumor size (≤4 vs. >4 cm) and cancer-specific survival in patients with resectable NSCLC without nodal involvement (*P* < 0.001); **b** the relationship between tumor location [left lower lobe (LLL) vs. non-LLL] and cancer-specific survival in patients with resectable NSCLC and nodal involvement (*P* = 0.042)

We performed a sub-analysis of NSCLC patients stratified in terms of nodal status. The relationship between the clinicopathologic characteristics and CSS in patients with or without lymph node metastasis was examined (Table 3). The 5-year CSS rate of node-negative patients was 73.8%. Univariate analysis indicated that gender (*P* = 0.022), tumor location (upper vs. non-upper, *P* = 0.024; LLL vs. non-LLL, *P* = 0.038), T category (*P* = 0.001), pleural invasion (*P* = 0.029), tumor size (*P* < 0.001), and pathology (*P* = 0.013) were statistically associated with CSS. However, multivariate analysis demonstrated that the only independent risk predictor for CSS was a tumor size of >4 cm (HR: 1.889, 95% CI 1.324–2.696, *P* < 0.001, Fig. 1a). In node-positive patients, the 5-year CSS rate was 40.8%, and the presence of LLL tumors was the only factor significantly associated with unfavorable CSS (HR: 1.528, 95% CI 1.015–2.301, *P* = 0.042, Fig. 1b).

**Table 3:** Univariate analysis for NSCLC patients with or without lymph node metastasis

Characteristic	Node-negative patients (pN0)		Node-positive patients (pN1–2)	
	HR (95% CI)	*P* ^a^	HR (95% CI)	*P* ^a^
Sex (men vs. women)	0.617 (0.409–0.932)	0.022	1.085 (0.761–1.546)	0.653
Age (≤60 vs. >60 years)	1.367 (0.959–1.949)	0.084	1.004 (0.713–1.413)	0.983
Tumor location				
(Left vs. right lobe)	0.980 (0.679–1.414)	0.913	0.857 (0.611–1.202)	0.372
(Upper vs. lower lobe)	1.505 (1.055–2.146)	0.024	1.132 (0.812–1.578)	0.465
(Non-LLL vs. LLL)	1.554 (1.024–2.357)	0.038	1.528 (1.015–2.301)	0.042
T category (T1 vs. T2 vs. T3)	1.794 (1.284–2.507)	0.001	1.025 (0.694–1.515)	0.901
N category (N1 vs. N2)	NA	NA	1.049 (0.725–1.518)	0.801
Pathology (AC vs. SCC vs. others)	1.357 (1.066–1.727)	0.013	0.943 (0.732–1.213)	0.646
Histology grade (G1 vs. G2 vs. G3)	1.258 (0.994–1.592)	0.056	1.023 (0.797–1.314)	0.857
Tumor size (≤4 vs. >4 cm)	1.889 (1.324–2.696)	<0.001	1.296 (0.924–1.816)	0.133
Pleural invasion (no vs. yes)	1.857 (1.064–3.242)	0.029	0.877 (0.505–1.525)	0.643
Resection (lobectomy vs. bilobectomy)	1.590 (0.774–3.265)	0.207	1.194 (0.699–2.040)	0.517
Adjuvant chemotherapy (yes vs. no)	1.490 (0.902–2.461)	0.120	1.241 (0.888–1.735)	0.207

*NA* not available. Other abbreviations as in Table  2

^a^Cox proportional hazards regression analysis (forward stepwise)

### The relationship between tumor location and clinicopathological factors

As shown in Table 4, the clinicopathologic characteristics were compared between LLL and non-LLL tumors. After the patients were dichotomized into node-positive and node-negative groups, we found that LLL tumors associated with large tumor size (*P* < 0.001) and high histological grade (*P* = 0.023) in node-negative but not node-positive patients. Irrespective of nodal status, the association between LLL tumors and a smaller number of dissected MLNs was significant (*P* < 0.001). There were also significantly fewer dissected MLNs in left-sided than in right-sided tumors [node-negative: 5 (range 3–21) vs. 9 (range 3–44), *P* < 0.001; node-positive: 5 (range 3–17) vs. 9 (range 3–38), *P* < 0.001]. However, the difference between the number of MLNs dissected in upper and non-upper tumors was not significant [node-negative: 7 (range 3–44) vs. 7 (range 3–29), *P* = 0.085; node-positive: 7 (range 3–38) vs. 7 (range 3–36), *P* = 0.772]. No significant differences in sex, age, T category, TNM stage, pathology, or pleural invasion were observed in any subgroups. However, in node-positive cases, more patients with LLL tumors than those with non-LLL tumors received adjuvant chemotherapy (*P* = 0.050), whereas no significant difference existed in node-negative patients (*P* = 0.063).

**Table 4:** Relationship between tumor location and clinicopathologic factors of NSCLC patients

Characteristic	Node-negative patients (pN0)			Node-positive patients (pN1–2)		
	LLL (*n* = 66)	Non-LLL (*n* = 324)	*P*	LLL (*n* = 41)	Non-LLL (*n* = 196)	*P*
Sex			0.702			0.264
Men	47 (71.2)	223 (68.8)		25 (61.0)	137 (69.9)	
Women	19 (28.8)	101 (31.2)		16 (39.0)	59 (30.1)	
Age			0.341			0.463
≤60 years	31 (47.0)	173 (53.4)		27 (65.9)	117 (59.7)	
>60 years	35 (53.0)	151 (46.6)		14 (34.1)	79 (40.3)	
T category			0.291			1.000^b^
T1	9 (13.6)	59 (18.2)		4 (9.8)	18 (9.2)	
T2	45 (68.2)	227 (70.1)		33 (80.5)	157 (80.1)	
T3	12 (18.2)	38 (11.7)		4 (9.8)	21 (10.7)	
N category			NA			0.170
N1	0	0		15 (36.6)	51 (26.0)	
N2	0	0		26 (63.4)	145 (74.0)	
TNM stage			0.445^c^			0.081^d^
Ia/b	63 (95.5)	299 (92.3)		0	0	
IIa/b	3 (4.5)	25 (7.7)		15 (36.6)	46 (23.5)	
IIIa	0	0		26 (63.4)	150 (76.5)	
Histology			0.260			1.000^b^
AC	30 (45.5)	183 (56.5)		26 (63.4)	124 (63.3)	
SCC	27 (40.9)	105 (32.4)		10 (24.4)	49 (25.0)	
Others	9 (13.6)	36 (11.1)		5 (12.2)	23 (11.7)	
Histology grade			0.023			0.256^b^
G1	14 (21.2)	68 (21.0)		2 (4.9)	19 (9.7)	
G2	13 (19.7)	117 (36.1)		10 (24.4)	66 (33.7)	
G3	39 (59.1)	139 (42.9)		29 (70.7)	111 (56.6)	
Tumor size			<0.001			0.801
≤4 cm	29 (43.9)	230 (71.0)		27 (65.9)	125 (63.8)	
>4 cm	37 (56.1)	94 (29.0)		14 (34.1)	71 (36.2)	
Pleural invasion			0.343			1.000^b^
Yes	57 (86.4)	264 (81.5)		37 (90.2)	178 (90.8)	
No	9 (13.6)	60 (18.5)		4 (9.8)	18 (9.2)	
Adjuvant chemotherapy			0.063			0.050
Yes	9 (13.6)	78 (24.1)		30 (73.2)	111 (56.6)	
No	57 (86.4)	246 (75.9)		11 (26.8)	85 (43.4)	
No. of MLNs dissected^a^	4 (3, 17)	7.5 (3, 44)	<0.001	5 (3, 14)	8 (3, 38)	<0.001

Abbreviations as in Tables 2 and 3

^a^The data are presented as medians followed by the range in the parenthese and analyzed by Mann-Whitney *U* test; other values are presented as the number of patients followed by the percentage in the parentheses

^b^Fisher’s exact test

^c^Comparison between patients with stages Ia/b and IIa/b NSCLC

^d^Comparison between patients with stages IIa/b and IIIa NSCLC

## Discussion

This study demonstrated that both non-upper and LLL tumors were unfavorable factors in CSS of patients with resectable NSCLC, as indicated by univariate analysis. However, only LLL tumors retained significance by multivariate analysis. The underlying reasons for this finding are not entirely clear, but we speculate that the lymphatic channel differs between lobes, likely resulting in different prognoses. Lower lobe tumors have the tendency to spread more frequently than upper lobe tumors to the subcarinal station, the passage of the hilar lymph nodes that connects with the contralateral nodes [19]. Hence, contralateral mediastinal drainage from the non-upper tumors may be more common, leading to unfavorable prognoses [20]. Moreover, left lung tumors spread to both the contralateral and ipsilateral lymph nodes with the same frequency [21], and the rate of occult metastasis to the right upper mediastinal nodes in left-sided NSCLC reached 72.7% [22]. Nohl-Oser et al. [23] also demonstrated that the highest metastatic rate to the contralateral mediastinum was 40% in LLL tumors, compared with 22% in LUL and 7% in RLL tumors.

Interestingly, after stratification based on lymph node status, the unfavorable prognosis of LLL tumors was found to be affected by the presence or absence of nodal involvement. LLL tumors were independently associated with CSS only in patients with lymph node metastasis, whereas a tumor size of >4 cm is the only independent risk predictor in the node-negative subgroup. This finding is consistent with that in a previous report by Kudo et al. [13], in which an influence of tumor location on the prognosis of patients with lymph node metastasis was also identified. It is tempting to attribute the dismal outcome of LLL to the fact that the mediastinal lymph nodes are prone to metastasis. However, after calculating the mediastinal metastatic rate of LLL and non-LLL tumors in node-positive patients, we found that tumor location presented no association with the metastasis of the mediastinal lymph nodes. Therefore, the LLL tumor was an intrinsic prognostic factor in this subgroup of patients. The observed disparity may be attributed to the different rates of occult metastasis in the contralateral nodes between node-positive and node-negative patients. In a recent study, Sakao et al. [24] revealed that >50% of patients with ipsilateral mediastinal nodal involvement suffered from occult pN3 disease. LLL tumors may be easier to be understaged to pN3 disease in node-positive patients than in node-negative patients, leading to a more unfavorable prognosis.

Contralateral MLN dissection was not performed in the present study; thus, the uncertainty of the status of the contralateral MLNs casts doubt on our results. However, we performed a systematic MLN dissection using the approach described in Allen’s study [18], which has been recognized as a standard procedure for staging resected NSCLC. Significantly fewer MLNs were dissected in LLL tumors than in non-LLL tumors, potentially because the left upper mediastinal lymph nodes were not routinely dissected in left-sided tumors in the mediastinal lymphadenectomy procedure used. The results revealed that there was only a significant difference in the number of dissected MLNs between the left- and right-sided tumors and not between the upper and non-upper tumors.

In this study, LLL tumors were positively associated with large tumor size and high histology grade in node-negative patients. This association may be because the left heart shadow makes it difficult to find LLL tumors. Tumors of larger size and poor differentiation are more invasive and progressive than smaller and well-differentiated ones. The close association between tumor location, size, and differentiation may explain why LLL tumors without lymph node metastasis are associated with poor survival in univariate analysis but not in multivariate analysis. However, in node-positive patients, tumor size and differentiation were not significantly different between LLL and non-LLL tumors, making tumor location the only significant prognosticator.

The limitations of this study include its retrospective nature, the small sample size, and the long, 9-year time-span of the evaluated cases. The treatment strategy changed numerous times over these years, possibly rendering the evaluation of the association between tumor location and prognosis more complicated. In this study, more node-positive patients with LLL tumors received postoperative chemotherapy than those with non-LLL tumors. Nevertheless, their CSS remains far from satisfactory. Routine and standard adjuvant chemotherapy for node-positive patients was not performed until 2004, and this limitation makes a prospective study on multimodality therapy versus tumor location necessary.

## Conclusions

This study revealed that the presence of LLL tumors was a robust unfavorable prognostic factor in resected NSCLC patients. However, its role accounted for node-positive but not node-negative patients, potentially because tumors located in the LLL might be upstaged in node-positive patients. Further studies are needed to determine the optimal management strategy for this subgroup.

## Abbreviations

NSCLC: non-small-cell lung cancer
TNM: tumor-node-metastasis
LLL: left lower lobe
CSS: cancer-specific survival
MLNs: mediastinal lymph nodes
RUL: right upper lobe
RMLL: right non-upper lobe
LUL: left upper lobe
MRI: magnetic resonance imaging
HRs: hazard ratios
CI: confidence interval

## References

[1] Chen W, Zheng R, Zeng H, Zhang S (2015). The updated incidences and mortalities of major cancers in China, 2011. Chin J Cancer..

[2] Ferlay J, Soerjomataram I, Dikshit R, Eser S, Mathers C, Rebelo M (2015). Cancer incidence and mortality worldwide: sources, methods and major patterns in GLOBOCAN 2012. Int J Cancer.

[3] Strauss GM, Herndon JE, Maddaus MA, Johnstone DW, Johnson EA, Harpole DH (2008). Adjuvant paclitaxel plus carboplatin compared with observation in stage IB non-small-cell lung cancer: CALGB 9633 with the Cancer and Leukemia Group B, Radiation Therapy Oncology Group, and North Central Cancer Treatment Group Study Groups. J Clin Oncol.

[4] Douillard JY, Rosell R, De Lena M, Riggi M, Hurteloup P, Mahe MA (2008). Impact of postoperative radiation therapy on survival in patients with complete resection and stage I, II, or IIIA non-small-cell lung cancer treated with adjuvant chemotherapy: the adjuvant Navelbine International Trialist Association (ANITA) Randomized Trial. Int J Radiat Oncol Biol Phys.

[5] Delaney C, Frank S, Huang RS (2015). Pharmacogenomics of EGFR-targeted therapies in non-small cell lung cancer: EGFR and beyond. Chin J Cancer..

[6] Rami-Porta R, Crowley JJ, Goldstraw P (2009). The revised TNM staging system for lung cancer. Ann Thorac Cardiovasc Surg..

[7] Iwasaki A, Shirakusa T, Enatsu S, Maekawa S, Yoshinaga Y, Yoneda S (2005). Is T2 non-small cell lung cancer located in left lower lobe appropriate to upstage?. Interact CardioVasc Thorac Surg.

[8] Ichinose Y, Kato H, Koike T, Tsuchiya R, Fujisawa T, Shimizu N (2001). Completely resected stage IIIA non-small cell lung cancer: the significance of primary tumor location and N2 station. J Thorac Cardiovasc Surg.

[9] Ou SH, Zell JA, Ziogas A, Anton-Culver H (2007). Prognostic factors for survival of stage I nonsmall cell lung cancer patients: a population-based analysis of 19,702 stage I patients in the California cancer registry from 1989 to 2003. Cancer.

[10] Hayakawa K, Mitsuhashi N, Saito Y, Furuta M, Nakayama Y, Katano S (1996). Impact of tumor extent and location on treatment outcome in patients with stage III non-small cell lung cancer treated with radiation therapy. Jpn J Clin Oncol.

[11] Inoue M, Sawabata N, Takeda S, Ohta M, Ohno Y, Maeda H (2004). Results of surgical intervention for p-stage IIIA (N2) non-small cell lung cancer: acceptable prognosis predicted by complete resection in patients with single N2 disease with primary tumor in the upper lobe. J Thorac Cardiovasc Surg.

[12] Puri V, Garg N, Engelhardt EE, Kreisel D, Crabtree TD, Meyers BF (2010). Tumor location is not an independent prognostic factor in early stage non-small cell lung cancer. Ann Thorac Surg.

[13] Kudo Y, Saji H, Shimada Y, Nomura M, Usuda J, Kajiwara N (2012). Do tumours located in the left lower lobe have worse outcomes in lymph node-positive non-small cell lung cancer than tumours in other lobes?. Eur J Cardiothorac Surg.

[14] Whitson BA, Groth SS, Andrade RS, Habermann EB, Maddaus MA, D’Cunha J (2012). T1/T2 non-small-cell lung cancer treated by lobectomy: does tumor anatomic location matter?. J Surg Res.

[15] Stallard J, Loberg A, Dunning J, Dark J (2010). Is a sleeve lobectomy significantly better than a pneumonectomy?. Interact CardioVasc Thorac Surg.

[16] De Zoysa MK, Hamed D, Routledge T, Scarci M (2012). Is limited pulmonary resection equivalent to lobectomy for surgical management of stage I non-small-cell lung cancer?. Interact CardioVasc Thorac Surg.

[17] Mountain CF, Dresler CM (1997). Regional lymph node classification for lung cancer staging. Chest.

[18] Allen MS, Darling GE, Pechet TT, Mitchell JD, Herndon JE, Landreneau RJ (2006). Morbidity and mortality of major pulmonary resections in patients with early-stage lung cancer: initial results of the randomized, prospective ACOSOG Z0030 trial. Ann Thorac Surg.

[19] Takizawa T, Terashima M, Koike T, Akamatsu H, Kurita Y, Yokoyama A (1997). Mediastinal lymph node metastasis in patients with clinical stage I peripheral non-small-cell lung cancer. J Thorac Cardiovasc Surg.

[20] Iwasaki A, Shirakusa T, Miyoshi T, Hamada T, Enatsu S, Maekawa S (2006). Prognostic significance of subcarinal station in non-small cell lung cancer with T1-3 N2 disease. Thorac Cardiovasc Surg.

[21] Bignall JR, Moon AJ (1955). Survival after lung resection for bronchial carcinoma. Thorax.

[22] Anami K, Yamashita SI, Yamamoto S, Chujo M, Tokuishi K, Moroga T (2013). Contralateral mediastinal lymph node micrometastases assessed by video-assisted thoracoscopic surgery in stage I non-small cell left lung cancer. Eur J Cardiothorac Surg.

[23] Nohl-Oser HC (1972). An investigation of the anatomy of the lymphatic drainage of the lungs as shown by the lymphatic spread of bronchial carcinoma. Ann R Coll Surg Engl.

[24] Sakao Y, Miyamoto H, Oh S, Takahashi N, Sakuraba M (2007). Clinicopathological factors associated with unexpected N3 in patients with mediastinal lymph node involvement. J Thorac Oncol..

